# Fault-Tolerant and Data-Intensive Resource Scheduling and Management for Scientific Applications in Cloud Computing

**DOI:** 10.3390/s21217238

**Published:** 2021-10-30

**Authors:** Zulfiqar Ahmad, Ali Imran Jehangiri, Mohammed Alaa Ala’anzy, Mohamed Othman, Arif Iqbal Umar

**Affiliations:** 1 Department of Computer Science and Information Technology, Hazara University, Mansehra 21300, Pakistan; zulfiqarahmad@hu.edu.pk (Z.A.); arifiqbalumar@yahoo.com (A.I.U.); 2 Department of Communication Technology and Networks, Universiti Putra Malaysia (UPM), Serdang 43400, Malaysia; m.alanzy.cs@gmail.com; 3 Laboratory of Computational Science and Mathematical Physics, Institute for Mathematical Research (INSPEM), Universiti Putra Malaysia (UPM), Serdang 43400, Malaysia

**Keywords:** scientific workflows, scheduling, fault-tolerant, Montage, clustering

## Abstract

Cloud computing is a fully fledged, matured and flexible computing paradigm that provides services to scientific and business applications in a subscription-based environment. Scientific applications such as Montage and CyberShake are organized scientific workflows with data and compute-intensive tasks and also have some special characteristics. These characteristics include the tasks of scientific workflows that are executed in terms of integration, disintegration, pipeline, and parallelism, and thus require special attention to task management and data-oriented resource scheduling and management. The tasks executed during pipeline are considered as bottleneck executions, the failure of which result in the wholly futile execution, which requires a fault-tolerant-aware execution. The tasks executed during parallelism require similar instances of cloud resources, and thus, cluster-based execution may upgrade the system performance in terms of make-span and execution cost. Therefore, this research work presents a cluster-based, fault-tolerant and data-intensive (CFD) scheduling for scientific applications in cloud environments. The CFD strategy addresses the data intensiveness of tasks of scientific workflows with cluster-based, fault-tolerant mechanisms. The Montage scientific workflow is considered as a simulation and the results of the CFD strategy were compared with three well-known heuristic scheduling policies: (a) MCT, (b) Max-min, and (c) Min-min. The simulation results showed that the CFD strategy reduced the make-span by 14.28%, 20.37%, and 11.77%, respectively, as compared with the existing three policies. Similarly, the CFD reduces the execution cost by 1.27%, 5.3%, and 2.21%, respectively, as compared with the existing three policies. In case of the CFD strategy, the SLA is not violated with regard to time and cost constraints, whereas it is violated by the existing policies numerous times.

## 1. Introduction

Cloud computing is a distributed and large-scale computing environment. It provides a pool of virtualized and dynamic computing services [1]. These services are delivered by the cloud environment in a subscription-based environment. Cloud services are highly scalable in nature and are provided to the customers dynamically and delivered transparently and not by manual means [2]. Services are provided to the external customers with a significantly high Internet speed on an on-demand basis with the computing architecture of three main services: “Infrastructure as a Service” (IaaS), “Platform as a Service” (PaaS) and “Software as a Service” (SaaS) [3,4]. Considering the cloud resources and services, large numbers of organizations use cloud environments to maximize their performance with a better Quality of Service (QoS) and system performance [5,6,7].

Cloud services in terms of usage are broadly categorized into scientific applications and business models [8]. Large-scale scientific applications such as Montage [9] and CyberShake [10] are often structured as scientific workflows. These applications are used and developed by scientists and need a high computational and storage power for their evaluation and solutions [11,12]. The major fields of scientific applications include: earthquake science, astronomy, gravitational physics and biology [13]. For instance, Montage [9] is one of the real-time scientific applications belonging to the field of astronomy. In Montage, input images are evaluated to obtain desired mosaics. It is a highly data-intensive workflow as it processes high-definition input images. The input images are obtained from a region of the sky by the astronomer for the purpose of obtaining the desired mosaics. The size of the desired mosaic is characterized by the square degree [14]. For example, in the 1-, 2-, and 4-degree square workflows in Montage, there are 203, 732 and 3027 application tasks, respectively. If a 4-degree-square Montage workflow is considered, it consists of 3027 application tasks with a runtime of 85 CPU hours with a cost of USD 9 when it is running on a single processor. Cloud computing provides the availability of a large number of resources with the most affordable price and a well-defined method for dynamically obtaining and releasing resources [11,15,16]. Therefore, for the evaluation of scientific data in an efficient and reliable manner, cloud computing is one of the most prominent platforms [17,18,19]. 

For the performance of workflow executions on target resources, there are two main workflows management strategies: (a) Pegasus WMS (Workflow Management System), and (b) Heterogeneous Event Management Middleware (HEMM). WMS was presented in [18,20]. In each workflow management strategy, it is supposed that when a task is allocated to a resource, the resource starts the execution after accepting the task. However, the factors such as: (a) dependencies between the tasks, (b) reliability, (c) scheduling policies, (d) QoS assurance, and (e) fault-tolerant mechanisms may be deployed in a system [21]. Due to such factors there is a risk of performance degradation [14]. Similarly, some of the workflows are too large and need to be moved from one node to another, resulting in significantly expensive data movement [22]. Moreover, there are some special characteristics of scientific workflows. These characteristics include the tasks of scientific workflows that are executed in terms of integration, disintegration, pipeline, and parallelism, and thus require special attention to task management and data-oriented resource scheduling and management [23]. The tasks executed during pipeline are considered as bottleneck executions, the failure of which result in the wholly futile execution, which requires a fault-tolerant-aware execution. The tasks executed during parallelism require similar instances of cloud resources, and thus, cluster-based execution may upgrade the system performance in terms of make-span and execution cost. [24,25]. All these challenges lead to the need for effective and well-defined workflows scheduling strategy with cluster-based, fault-tolerant mechanisms.

In this research work, a systematic approach is used to find the solutions to all the above-mentioned challenges. Therefore, a cluster-based, fault-tolerant and data-intensive (CFD) resource scheduling and management strategy for scientific applications in cloud computing is proposed. The main contributions of this work are provided below:

- A cluster-based, fault-tolerant and data-intensive (CFD) resource scheduling and management strategy for scientific applications in a cloud environment is proposed in this research work. The CFD strategy is elaborated through multiple elements, e.g., user and application interface, Workflow Admission, Workflow Mapper, Workflow Scheduler, and Workflow Engine.
- There are four core components of the CFD strategy, e.g., (a) Workflow Admission, (b) Workflow Mapper, (c) Workflow Scheduler, and (d) Workflow Engine. These components convert scientific data submitted by one or more users into scientific workflows and assign them to the required resources for execution.
- A selective reclustering-based, fault-tolerant mechanism [26,27,28,29] is provided to the CFD strategy.
- The CFD strategy is evaluated through WorkflowSim [30,31], while considering the performance evaluation parameters, e.g., execution time, cost, budget, deadline and SLA violation.
- In order to show the efficiency of the CFD strategy, the Montage [14] scientific workflow is executed and compares the simulation results with three well-known heuristic scheduling policies: (a) “Minimum Completion Time” (MCT) [32], (b) “Max-min” [22], and (c) “Min-min” [22] scheduling policies. The simulation results reflect that the proposed CFD strategy outperforms the current solutions.

The remainder of the paper is organized as follows: Section 2 presents the related work; Section 3 presents the System Model and Design; Section 4 provides in detail the experiments, results and discussions; and Section 5 concludes the work.

## 2. Related Work

The related work is reviewed thoroughly in terms of scientific workflow scheduling and fault-tolerant techniques. The basic characteristics of scientific workflows were explored. At the very beginning, a study was conducted on five basic realistic scientific applications [13]. These workflows included CyberShake (earthquake science), Montage (astronomy), Laser Interferometer Gravitational Wave Observatory (LIGO) (gravitational physics), Epigenomics (biology) and SIPHT (biology). The study provides the basic information, structure and behavior of each workflow in terms of its execution. A comprehensive characterization of each application with structural, computational and data requirements was pointed out. It was also noted that the scientific workflows had some special structural properties in terms of pipeline execution, data aggregation, data parallelism, data distribution and data redistribution. 

In order to improve the workflow completion time and efficient utilization of available resources, a scheduling strategy, Adaptive Data Aware Scheduling (ADAS), was presented [33]. The ADAS strategy is an integrated data and task management strategy in a cloud environment for workflow applications that uses two stages. In the first stage, which is called the “setup stage”, the cluster is created and in the second stage, which is called the “run stage”, the workflow is executed. The presented work lacks the fault-tolerant mechanism. The scheduling was one of the major components for the execution of scientific workflows and its importance could not be denied at any stage of execution. Thus, “Fault-Tolerant Workflow Scheduling (FTWS) Using Spot Instances on Clouds”, a scheduling algorithm for the execution of workflows which is robust against the performance variations was proposed in [34]. The scheduling algorithm schedules tasks by using two cloud pricing models i.e, on-demand and spot instances pricing models. These models are specifically designed to reduce the cost of execution within the constraint of deadlines. The work proposed in [34] is only for generic types of workflow and is mostly used for business applications, such as business models for Amazon, and lacks particularity for scientific applications. 

In [35], three scheduling algorithms were developed to provide resources for the ensembles of workflows and their scheduling on the cloud, within the limits of deadlines and budget constraints. The first scheduling algorithm is Dynamic Provisioning Dynamic Scheduling (DPDS), which is an online scheduling algorithm that schedules tasks and provides resources at runtime. It consists of two procedures: (a) the scheduling procedure and (b) the provisioning procedure. The second scheduling algorithm is Workflow-aware DPDS (WA-DPDS) which extends the DPDS, as well as initiates a Workflow Admission module. The third and last scheduling algorithm of the work presented in [35] is Static Provisioning Static Scheduling (SPSS) which is the static version of WA-DPDS [35]. It creates a scheduling and provisioning plan before running any workflow tasks. In other words, SPSS only starts those workflows for executions that can be completed within budget and deadline constraints, while the rest of the workflows are rejected without starting their execution. The algorithm is specifically designed for an ensemble of workflows which are not suitable for the execution of heterogeneous types of workflows, i.e., when different types of workflows need to be executed and not generally applicable for all types of workflows independently. Fault tolerance parameters are also not included in the presented work.

Aside from the above-mentioned scheduling algorithms, there are various heuristic methods, such as “Minimum Completion Time” (MCT) [36], “Maximum-minimum” (Max-min) [37] and “Minimum-minimum” (Min-min) [38]. These methods are used for the scheduling of independent tasks of scientific applications [32]. In MCT, the completion time of each task is calculated and tasks with a shorter completion time are allocated first. In Max-min, firstly the large tasks are executed leading to delay in tiny tasks, while in Min-min, the small tasks are executed first, leading to a delay in the larger tasks. The majority of the tasks in the scientific workflows are numerous and represent large amounts of computations, as well as data [39]. Therefore, load balancing is one of the main issues in scheduling techniques, specifically for the execution of such types of scientific workflows through Workflow Management System because, when load balancing is conducted in scheduling techniques, the Dependency Imbalance and Runtime Imbalance occur. To overcome such deficiencies, the Balance Task Clustering (BTC) Technique for scientific workflows is presented in [40]. There were three main goals which were achieved in BTC. Firstly, a series of metrics were proposed that revealed the internal structure of the workflow which supported the reducing runtime and dependency imbalance. Secondly, a concept of family and neighboring task clustering was used as there was a strong connection between parents, children, and siblings. Finally, the balancing method and quantitative metrics were analyzed, i.e., workflow imbalance problems were characterized by metrics and, by the comparison of the relative values, a balancing method was selected. The work presented in [40] focused on load balancing, while the rest of the factors of scheduling (e.g., data management, task assignment, time and cost) remained intact. The overhead of metrics and dependency analysis could not be denied as there were numerous numbers of short-running tasks in the workflows system. Furthermore, if we apply the fault tolerance methods on the given technique, there were chances of reducing the performance with respect to workflow completion time. Similarly, a load-balancing technique was proposed concerning only time, while the cost of tasks was not considered at any stage. Moreover, the authors did not mention whether the BTC technique was an independent technique or whether it was intended to be used with another model as a mere assertion of system architecture. Moreover, the workflow system model was unable to consider all the factors, such as cost, data management, task assignment, and fault tolerance.

In [41], a data-aware scheduling strategy referred to as “Enhanced Data-oriented Scheduling strategy with Dynamic clustering fault-tolerant technique” (EDS-DC) was given. The strategy was particularly developed for applications related to scientific work. The authors used WorkflowSim [30] as a simulation environment and contended that their results demonstrated significant improvements. The EDS-DC strategy did not consider the appropriate procedure of workflow submission in relation to the generation of results. In [28], a cluster-based, fault-tolerant strategy referred to as “Fault-tolerant Clustering” (FTC) for scientific applications was presented. In FTC, the authors categorized the failures as task failures and job failures. The authors further contended that a job was a cluster of multiple tasks. When a single cluster of tasks fails then it was called a job failure. However, when a single task or combination of tasks in a cluster failed to execute tasks, this was called a task failure. In task failure, the failure of the overall job is not necessary. The work presented provided three methods as fault-tolerant mechanisms and solved the problem of the environment with faults. The first method was Dynamic Clustering (DC) which adjusted the clustering factor dynamically and according to the failure rate of the detected tasks. The second method was Selective Re-Clustering (SR) that, within a job, retried the failed tasks. The last method was Dynamic Re-Clustering (DR) that combined the first two methods. Not only did this method adjust the clustering factor dynamically according to the failure rate of detected tasks but it also retried the failed tasks of a job. The limitation of this work was that it was deficient with respect to the cost and time parameters.

In [42], four approaches were presented in order to automate the mechanism of the assignment of high-volume workloads to cloud computing resources. These approaches were: deep Q networks (DQN), reinforcement learning (RL), deep reinforcement learning combined with LSTM (DRL-LSTM) and recurrent neural network long short-term memory (RNN-LSTM). The main aim of these approaches was to reduce the task waiting time and resource consumption. In [43], a multi-objective simulated annealing (MOSA) strategy was designed for the secure allocation of tasks on the cloud and fog nodes. The allocation was made on the basis of multiple goals including access level and client demand. In [44], the authors extended the multi-objective scheduling policy, Harris hawks optimizer (HHO), by presenting the elite learning, Harris hawks optimizer (ELHHO). A scientifically intelligent method, “elite opposition-based learning”, was used to modify the existing HHO policy in order to solve the multi-objective scheduling problem. The authors claim that the presented ELHHO strategy satisfied the quality of service regarding minimizing schedule length, execution cost and maximizing resource utilization. 

In [45], a communication enhancement tool was proposed named BurstFlow. It was used to improve communication between Big Data Stream Processing applications with the edges of the Internet. This tool was based on cloud infrastructure. In the BurstFlow, an introduction of micro-batch size adjustment was made as per the required computation and communication time. An adaptive data partition policy was also introduced in BurstFlow that distributed the incoming data streams to the available resources based on the capacities of memory and CPU. 

A comprehensive comparison of literature review is shown in Table 1. 

The above-mentioned literature review reflects that when tasks of real-time scientific workflows are executed, they pertain to several issues and require respective solutions. The problem formulation of the proposed research work is described as below:

Suppose there are “n” number of tasks (T1, T2, T3, …, Tn) in a scientific workflow executed with “i” number of levels (L1, L2, L3, …, Li) on “j” number of given resources (R1, R2, R3, …, Rj):

- Based on the data intensiveness and special characteristics of scientific workflows, there are various tasks from T_1_, T_2_, T_3_, …, T_n_ with diverse requirements of resources from R_1_, R_2_, R_3_, …, R_j_ at multiple levels, L_1_, L_2_, L_3_, …, L_i_. Therefore, there should be efficient data-intensive resource scheduling.
- At several levels, there are multiple tasks from T_1_, T_2_, T_3_, …, T_n_ which are executed in parallel and require similar instances of cloud resources. Therefore, cluster-based scheduling will upgrade the system performance in terms of make-span and execution cost.
- Several tasks from T_1_, T_2_, T_3_, …, T_n_ are executed at bottleneck node/level, the failure of which makes the whole execution fruitless. Therefore, they require a fault-tolerant mechanism.

The proposed cluster-based, fault-tolerant and data-intensive (CFD) scheduling strategy addresses the above-mentioned issues in an efficient way. The proposed CFD strategy is a multi-criteria optimization technique that considers the two significant criteria of scientific workflow task management and scheduling, i.e., the data-intensiveness and fault tolerance. Both of these components are worthwhile to schedule and manage the tasks of scientific workflows in terms of make-span, execution cost and SLA violations. 

## 3. System Design and Model

This research work proposes a cluster-based, fault-tolerant and data-intensive (CFD) resource scheduling and management for scientific applications in a cloud environment with the following assumptions:

- Each job consists of multiple tasks of a similar nature.
- Resources are obtained from the cloud in terms of infrastructure as a service and are managed through the workflow management system.
- Clustering is a term used for the integrating of similar tasks in a group for their execution.
- The average budget and deadline for each type scientific workflow is considered as per its execution cost and time.

**Figure 1:**
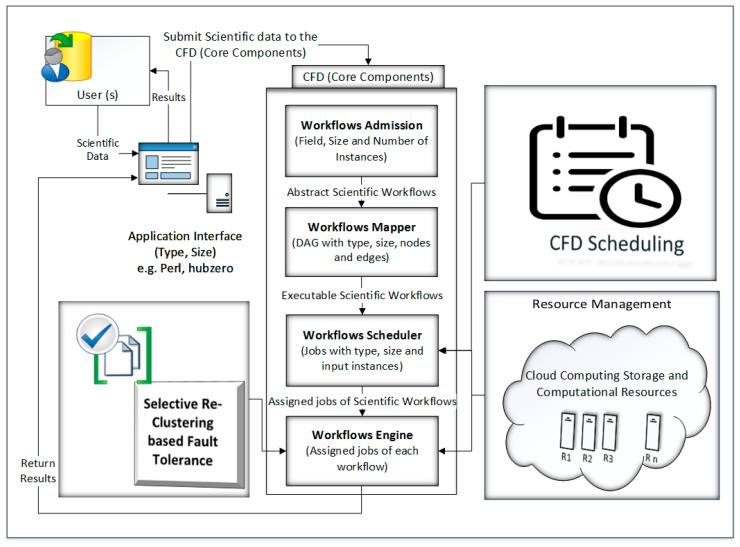
CFD strategy. The components of the CFD strategy are described below.

In the proposed CFD strategy, one or more users submit scientific data for execution through an application interface. The overall model of proposed work is shown in Figure 1. The cloud resources are obtained in terms of infrastructure as a service and managed through resource management. The core components of the CFD strategy are: (a) Workflow Admission, (b) Workflow Mapper, (c) Workflow Scheduler, and (d) Workflow Engine. Initially, the scientific data are sent to Workflow Admission, which converts the scientific data into abstract scientific workflows and then sends it to the next element, i.e., Workflow Mapper. Workflow Mapper converts the abstract scientific workflows into the executable scientific workflows which are then submitted to Workflow Scheduler. Workflow Scheduler converts the executable scientific workflows into jobs and then assigns them to the required resources. The execution process is conducted by Workflow Engine. The Workflow Engine executes the assigned jobs of each workflow as received from Workflow Scheduler with a cluster-based, fault-tolerant mechanism. After the execution of workflows in the form of jobs by Workflow Engine, the result of each workflow is returned to the respective user through the application interface. 

### 3.1. User

A user is an entity that can be an organization or a person. If a user is a person, then he/she can submit scientific data for its execution and evaluation. If the user is an organization, then it can obtain scientific data from a person and submit it for execution and evaluation. Users can submit single types of scientific data, as well as multiple types of scientific data. Normally, scientific data is related to realistic scientific applications, such as CyberShake, Montage, SIPHT and Epigenomics [13]. The number of users may be one or more than one and the single user, as well as multiple users, can submit a single type of scientific workflow, as well as multiple types of scientific data.

### 3.2. Application Interface 

The Application Interface provides an interface between the user and the CFD model. The user submits scientific data for execution and evaluation through the application interface. For the submission of scientific data to the CFD scheduling system, the Application Interface submits data to the next element of the model, i.e., Workflow Admission, with characteristics like the input, output, size and type of scientific data. Examples of Application Interfaces are Perl and hub-zero [20]. The Application Interface provides an interface to one or more than one user, as well as for one or more than one scientific workflow to the CFD scheduling system. 

### 3.3. Workflow Admission 

The Workflow Admission receives scientific data through an application interface which is generated by the scientists for the execution and evaluation of scientific applications. The Workflow Admission generates an abstract scientific workflow for scientific data. An abstract scientific workflow is a workflow which is in the form of Directed Acyclic Graph (DAG) and related to a single field of science. This type of workflow is submitted by a single user with one or more jobs/inputs producing a single result/output. The Workflow Admission simply receives scientific data which may consist of one or more than one field as per specified by the user, separate them into their respective scientific workflows, make their DAG and submit for the next element of the model, i.e., Workflow Mapper. As there may be more than one user, it is the responsibility of Workflow Admission to generate an abstract scientific workflow for each user and with its respective field of science specified by the user.

Algorithm 1 shows the overall procedure for Workflow Admission. The user submits scientific data, which contain specific fields of scientific data, e.g., astronomy and biology. They also contain the properties of size and the number of instances of scientific data. Workflow Admission simply checks whether the scientific data contain a single field/type of scientific data or have multiple types. If scientific data contains a single field/type, then Workflow Admission analyzes the number of nodes and dependencies between the nodes and submits them to the next core element of the CFD as an abstract scientific workflow in the form of Directed Acyclic Graph (DAG). If scientific data have multiple fields of data then Workflow Admission separates each field and analyzes the number of nodes and dependencies between the nodes of each field, then submitting it to the next core element of the CFD as an abstract scientific workflow in the form of DAG.
**Algorithm 1:** Workflow Admission
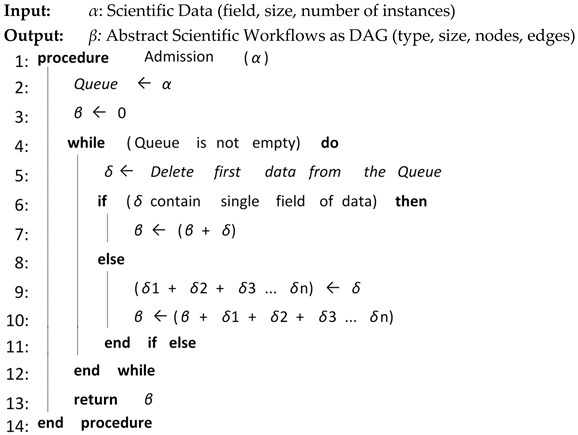


### 3.4. Workflow Mapper

Workflow Mapper receives one or more separate abstract workflows from Workflow Admission and generates executable workflows for each abstract workflow. At this stage the parameters of each workflow are the type of workflow, its size, input instances and output. The basic purpose of Workflow Mapper is to create such a workflow consisting of data in the form of jobs/inputs with requirements for computation and storage resources, so that these jobs/inputs can be submitted for the next elements of the CFD, e.g., Workflow Scheduler, to schedule them. 

Algorithm 2 shows the overall procedure for Workflow Mapper. Workflow Mapper receives abstract scientific workflow from Workflow Admission in the form of DAG. It converts the abstract scientific workflow into an executable scientific workflow by finding the jobs/tasks with the respective required resources of the abstract scientific workflow. Then, Workflow Mapper submits all the jobs of the respective scientific workflow to the next element for assignment of resources.
**Algorithm 2:** Workflow Mapper
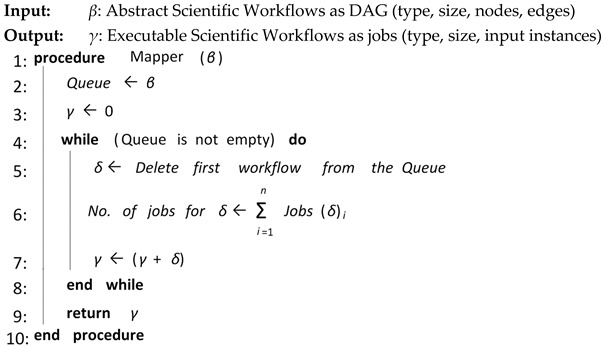


### 3.5. Workflow Scheduler 

Workflow Scheduler receives jobs/inputs from Workflow Mapper of each workflow and schedules them by assigning required resources. It is pertinent that the resources are obtained from the cloud in terms of Infrastructure as a Service (IaaS) and then scheduled/managed distinctly through the CFD. Workflow Scheduler also converts the jobs into the task and then allocates resources. Resources are allocated in such a way that they have the best execution time for the cheapest cost. 

Algorithm 3 shows the overall procedure for Workflow Scheduler. Workflow Scheduler receives jobs of scientific workflow from Workflow Mapper. Then, it finds required resources for each job. If necessary, it also converts the job into multiple tasks. Then, Workflow Scheduler checks for the required resources. If available, the resources fulfill the requirements of all the jobs of executable scientific workflow, then, Workflow Scheduler assigns the jobs to the required resources with the best minimum execution time and cost. Finally, Workflow Scheduler submits the assigned jobs to the next core elements for execution.
**Algorithm 3:** Workflow Scheduler
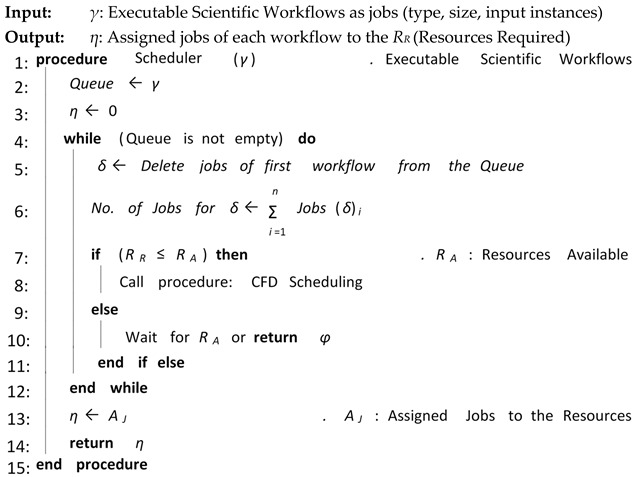


Algorithm 4 is a scheduling algorithm that represents the procedure of CFD scheduling. In this procedure, the list of tasks and resources are obtained and the mapped list of tasks to resources is returned. It assigns the best available resource to each task with a minimum data transfer time.
**Algorithm 4:** CFD Scheduling
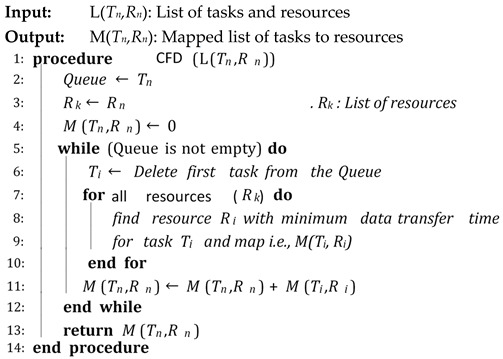


### 3.6. Workflow Engine 

Workflow Engine executes the jobs/tasks on their assigned resources as received from Workflow Scheduler. Workflow Engine also initiates the fault-tolerant mechanisms in such a way that if the execution of each job/task succeeds then it generates results and returns to the user through the application interface. If the execution of jobs/tasks is not succeeded or fails, then Workflow Engine initiates the fault-tolerant technique and retries or re-executes the failed tasks/jobs.

Algorithm 5 shows the overall procedure for Workflow Engine. Workflow Engine receives and assigns jobs/tasks to the required resources from Workflow Scheduler and executes them. If execution is successful then Workflow Engine generates results. If the execution of jobs/tasks fails, Workflow Engine initiates selective re-clustering based on a fault-tolerant technique. In our case we consider a 5% failure rate out of the total number of tasks and, as such, the fault-tolerant technique is initiated each time the workflow is submitted.
**Algorithm 5:** Workflow Engine
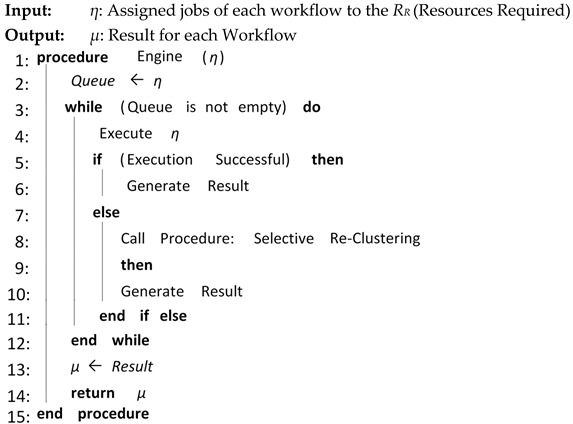


Algorithm 6 is a fault tolerant algorithm that represents the procedure of the fault-tolerant technique, Selective Re-clustering. Selective Re-clustering is a clustering technique that finds all the failed tasks from each job, clusters them and then re-executes that cluster containing the failed tasks. The input to the algorithm is a list of available resources and failed jobs. The algorithm returns the mapped list of failed jobs to the required resources for execution.
**Algorithm 6:** Selective Re-Clustering
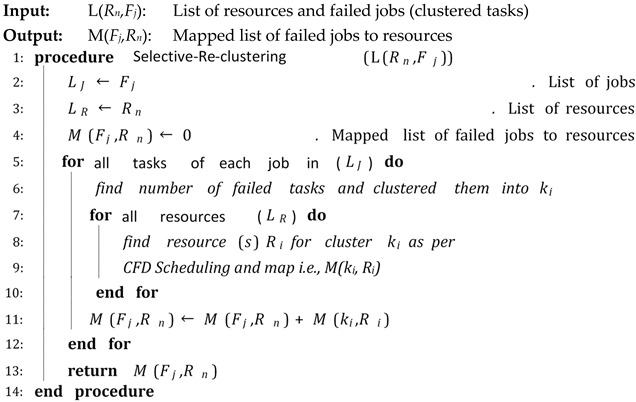


The proposed CFD (cluster-based, fault-tolerant and data-intensive) strategy is a component-based resource scheduling and management strategy for scientific applications in cloud environments. The major components of the CFD strategy are Workflow Admission, Workflow Mapper, Workflow Scheduler, CFD scheduling, Workflow Engine, and a fault-tolerant mechanism. All these components work with stepwise mechanisms and are elaborated through algorithms. There are six algorithms in the article, out of which four algorithms are related to workflow management, one algorithm (Scheduling Algorithm) is related to workflow scheduling and one algorithm (Fault-tolerant Algorithm) is related to the provision of fault-tolerant mechanism. Algorithm 1 shows the overall procedure for the “Workflow Admission component” of the CFD strategy in which there is a single “while loop”; therefore, the complexity of Algorithm 1 is linear. Algorithm 2 shows the overall procedure for the “Workflow Mapper component” of CFD strategy, while Algorithm 3 shows the overall procedure for the “Workflow Scheduler component”. Both Algorithms 2 and 3 work with a single “while loop”, and thus bear linear time complexities. Algorithm 4 provides the scheduling process of the proposed CFD strategy. In Algorithm 4, there is a “for loop” inside the “while loop”. For each iteration of the “while loop”, the “for loop” runs “n” number of times. Therefore, the complexity of Algorithm 4 is exponential. Algorithm 5 shows the overall procedure for the “Workflow Engine” of the CFD strategy in which there is a single “while loop”; therefore, the complexity of Algorithm 5 is linear. Algorithm 6 provides the fault-tolerant mechanism of the proposed CFD strategy. In Algorithm 6, the nested “for loop” is used, and thus it has an exponential time complexity.

## 4. Experiment, Result and Discussion

### 4.1. Simulation Setup

The detailed description of the simulation environment regarding the resources and specifications of scientific workflows submitted by one of more users, is given below. 

**Resource modeling**

The simulation is performed in WorkflowSim [30], “a toolkit for simulating scientific workflows”. It is modified to support the fault-tolerant mechanism and scheduling policy. The WorkflowSim is a workflow-based simulation tool which is used to implement workflow scheduling and management techniques; however, the proposed CFD strategy, in terms of scheduling policy and fault-tolerant mechanisms, is not previously implemented in WorkflowSim. Therefore, the proposed CFD strategy is implemented in WorkflowSim to simulate a Montage [9,14,20,32] scientific workflow, which is one of the real-time scientific applications belonging to the field of astronomy. Space-shared resources are used with certain characteristics, such as budget and deadline. The remaining specifications are shown in Table 2 as below.

**Application modeling**

In our proposed scenario, one user submits the real-time scientific workflow in Montage [9,14,20,32] with 25, 50, 100 and 1000 tasks, respectively. Montage [9] is one of the real-time scientific applications belonging to the field of astronomy.

### 4.2. Performance Evaluation Parameters

A comprehensive detail of each performance evaluation parameter used in this article is described as below:

**Make-span**

Make-span is the time spent to execute a batch of jobs. In the context of scientific workflows, it is the overall time needed to execute the scientific workflow [34]. It is denoted by M and evaluated with the help of Equation (1):

(1) M=F.T−S.T 

where F.T denotes the finish time and S.T denotes the start time of scientific workflow execution.

**Deadline**

The deadline is the predefined execution time of a batch of jobs. In the context of scientific workflows, it is the predefined overall execution time for the execution of a scientific workflow [22]. It is denoted by D and can be evaluated with the help of Equation (2):

(2) D=Computation Time+Communication Time+Overhead

where the overhead is the additional time consumed during the re-execution of failed jobs/tasks of scientific workflow. 

**Cost**

Cost is the budget spent to execute a batch of jobs. In the context of scientific workflows, it is the overall budget needed for the execution of scientific workflows [34]. It is denoted by C and can be evaluated with the help of Equation (3):

(3) C=Cost on F.T−Cost on S.T 

where F.T denotes the finish time and S.T denotes start time. Similarly, the cost for each task is denoted by $C_{t}$ and is computed with the help of Equation (4):

(4) $$C_{t}=Processing\;Cost+Memory\;Cost+Storage\;Cost+Bandwidth\;Cost\;\;$$

**Budget**

The budget is the total monetary resources needed to execute a batch of jobs [22]. In the case of scientific workflows, it is the predefined cost needed for the execution of a scientific workflow. It is denoted by B and can be calculated with the help of Equation (5):

(5) B=Computation Cost+Communication Cost+Overhead 

where the overhead is the extra cost consumed when failed jobs/tasks of scientific workflow are re-executed. 

**SLA Violation**

Service Level Agreement (SLA) is violated when the cost is exceeds the predefined budget or when the make-span exceeds the predefined deadline [22]. Equations (6) and (7) represent the conditions for SLA violation [46]:

(6) $$SLAV=SLAV_{ITAH\;}$$

(7) $$SLAV=SLAV_{ICAB\;}$$

The SLAV represents SLA violation, $SLAV_{ITAH}$ represents SLA violation due to the increase in time per active hours and $SLAV_{ICAB}$ represents the increase in cost in SLA violation per active budget. 

### 4.3. Results and Discussion

One user is considered for simulation who submits the real-time scientific workflows Montage with 25, 50, 100 and 1000 tasks. The objective is to evaluate the CFD strategy by executing the real-time scientific workflow Montage. The execution cost, budget, make-span, deadline and the SLA violation are the performance evaluation parameters. 

**Figure 2:**
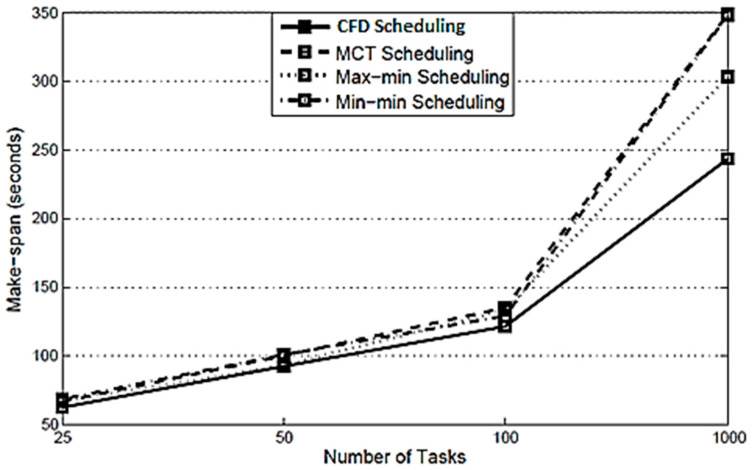
Comparison of CFD scheduling with MCT scheduling, Max min scheduling, and Min min scheduling in respect of make-span.

**Make-span:** The results, with regard to the make-span for the CFD strategy compared with the existing MCT, Max min and Min min scheduling are shown in Figure 2 which reflects that the make-span is the minimum for the proposed CFD strategy. Due to this, the CFD strategy finds the nearest available resource for each task and, as such, it reduces the make-span and cost, as compared with the other three scheduling policies.

**Figure 3:**
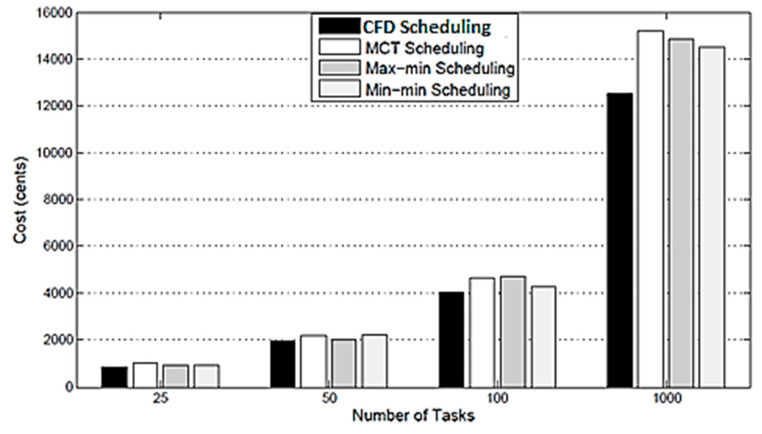
Comparison of CFD scheduling with MCT scheduling, Max min scheduling, and Min min scheduling in respect of cost.

**Cost:** The results with respect to cost of the CFD strategy compared with MCT, Max min and Min min scheduling are plotted in Figure 3, which reveals that in almost all the scheduling policies, the cost is the minimum for the proposed scheduling. Due to this, the CFD strategy finds the nearest available resource for each task and, as such, it reduces the make-span and cost compared with the other three scheduling policies.

**SLA Violation:** Table 3 shows the results of the CFD strategy along with the existing scheduling policies. In the case of the proposed CFD strategy, the SLA is not violated by time constraints or cost constraints. While for the other scheduling policies, it is violated several times. Due to this, the CFD strategy finds the nearest available resource for each task, and thus reduces make-span and cost, as compared with other three scheduling policies.

The proposed CFD strategy is a novel approach, as in the CFD strategy is a detailed process from scientific data submission to the generation of results, and component-based scenarios are provided. Each component is elaborated in detail using pseudo code. There are six algorithms along with detailed descriptions. The experiments are performed in Montage workflow, which is a scientific application related to the field of astronomy. The CFD strategy addresses the data and computes the intensiveness of tasks of scientific workflows with a cluster-based, fault-tolerant mechanism. The Montage scientific workflow is simulated. The results of the proposed CFD strategy are compared with three well-known heuristic scheduling policies: (a) MCT, (b) Max-min, and (c) Min-min. However, these heuristic scheduling policies are also equipped with the fault-tolerant mechanism, so that the comparison was made justly and fairly. The performance is comparable to these heuristic scheduling policies because the scientific workflows are scheduled and managed in terms of data-intensive tasks with a diverse nature. There is a large variety of scientific workflows tasks; they may be of huge size, normal size or of very small size. Therefore, in such circumstances, MCT, Max-min, and Min min are effective when considering the completion time, and the maximum- and minimum-sized nature of the tasks of scientific workflow. However, these policies do not consider the data-intensiveness of the tasks including the data transfer time. These policies are also not equipped with any fault-tolerant mechanisms in their original form. As such, the simulation results of the proposed CFD strategy are superior to the heuristic policies for scientific workflows. 

So far, as the question of comparison with any other recent strategy is concerned, the recent strategies have not specifically addressed the issues of scientific workflows management and scheduling by considering the data-intensiveness, tasks variety and bottleneck failures of the tasks of scientific workflows. However, in future studies, this work will be extended to include energy efficient scheduling and a fault-tolerant framework with multi-criteria components and comparisons will be made with recent strategies for each criterion component.

## 5. Conclusions

In this research work, a cluster-based, fault-tolerant and data-intensive (CFD) strategy for scientific applications in a cloud environment is proposed. The proposed CFD strategy provides a detailed process from scientific data submission to the generation of results with component-based scenarios. Each component is elaborated in detail with pseudo code. The experiments were performed in Montage workflow, which is a scientific application related to the field of Astronomy. In the proposed CFD strategy, one or more users submit scientific data for execution through an application interface. The cloud resources are obtained in terms of Infrastructure as a Service and managed through Resource Management. The core components of CFD strategy are: (a) Workflow Admission, (b) Workflow Mapper, (c) Workflow Scheduler, and (d) Workflow Engine. Initially the scientific data was sent to Workflow Admission which converts the scientific data into abstract scientific workflow and then sends it to the next element, i.e., Workflow Mapper. Workflow Mapper converts the abstract scientific workflows into the executable scientific workflows, which are then submitted to Workflow Scheduler. Workflow Scheduler converts the executable scientific workflows into jobs and then assigns them to the required resources. The execution process is conducted by Workflow Engine. Workflow Engine executes the assigned jobs of each workflow as received from Workflow Scheduler with a cluster-based, fault-tolerant mechanism. After the execution of workflows in the form of jobs at Workflow Engine, the result of each workflow is returned to the respective user through the application interface. The CFD strategy addresses the data and computes the intensiveness of tasks of scientific workflows with a cluster-based, fault-tolerant mechanism. The Montage scientific workflow is considered for the simulation, and the results of the CFD strategy were compared with three well-known heuristic scheduling policies: (a) MCT, (b) Max-min, and (c) Min-min. The simulation results show that the CFD strategy reduces the make-span by 14.28%, 20.37%, and 11.77%, respectively, when compared with the existing three policies. Similarly, the CFD reduces the execution cost by 1.27%, 5.3%, and 2.21%, respectively, as compared with the existing three policies. In the case of the CFD strategy, the SLA is not violated for time and cost constraints, whereas it is violated several times by the existing policies. 

In future work, this research work will be extended in order to propose an energy-efficient, fault-tolerant-based scheduling framework for scientific workflows in cloud computing.

## Figures and Tables

**Table 1:** Comparison of Related Work.

References	Scheduling Policy	Fault Tolerance Mechanism	Resource Management	Performance Parameters		Features	Limitations
				Time	Cost		
[13]	✘	✘	✘	✘	✘	Details of five realistic scientific workflows are given	No workflows management, scheduling and fault-tolerant techniques
[20]	Pegasus WMS	✘	✔	✘	✘	Provides WMS structure for scientific workflows	No fault-tolerant mechanism.
[28]	✘	FTC	✔	✘	✘	Provides fault-tolerant mechanism for scientific workflows	No scheduling and task management
[33]	ADAS	✘	✔	✔	✘	Improves workflow completion time	No fault tolerance and workflows management
[34]	FTWS	Check-pointing	✔	✘	✔	Schedule tasks using two pricing models, e.g., spot and on-demand instances	No method to reduce the make-span
[35]	DPDS, WA-DPDS & SPSS	✘	✔	✔	✔	Provide resources for cluster of workflows	No fault tolerance and workflow management
[40]	BTC	✘	✔	✔	✘	Provide load balancing mechanism	There is the overhead of metrics and dependency analysis
[41]	EDS-DC	Dynamic Clustering	✔	✔	✔	Scheduling and fault-tolerant mechanism for scientific workflows	No scientific workflows management

**Table 2:** The specifications of resources used for simulation.

No. VMs	Memory	BW	VM	Arch
1000 VMs	10,240 MB	10000 Mbps	Xen	X86
**OS**	**Cost per VM $/Hr**	**Memory Cost $/s**	**Storage Cost $/s**	**Data Transfer Cost $/s**
Linux	3.0 $/h	0.05 $/s	0.1 $/s	0.1 $/s

**Table 3:** Shows deadline, budget and SLA Violation.

Scientific Workflow	Scheduling Policy	Make-Span (s)	Cost (cents)	Deadline (s)	Budget (cents)	SLA Violation	
						For Time	For Cost
Montage-25	***CFD***	***62.742***	***844.418***	70.00	1000.00	***No***	***No***
	MCT	67.71	993.236			No	No
	Max-min	67.476	930.482			No	No
	Min-min	68.21	925.484			No	No
Montage-50	***CFD***	***92.556***	***1951.912***	95.00	2200.00	***No***	***No***
	MCT	100.912	2192.62			Yes	No
	Max-min	93.632	1993.05			No	No
	Min-min	101.882	2227.616			Yes	Yes
Montage-100	***CFD***	***121.256***	***4016.798***	130.00	4400.00	***No***	***No***
	MCT	135.08	4619.592			Yes	Yes
	Max-min	133.994	4725.948			Yes	Yes
	Min-min	129.318	4287.208			No	No
Montage-1000	***CFD***	***1544.662***	***42,547.402***	1600.00	45000.00	***No***	***No***
	MCT	1649.732	45,188.35			Yes	Yes
	Max-min	1603.578	44,875.44			Yes	No
	Min-min	1648.09	44,499.09			Yes	No

## Data Availability

The details of input data for numerical experiments that support the findings of this study are openly accessible in the Synthetic Workflow Generator Gallery at https://pegasus.isi.edu/workflow_gallery/ [14,20,32] (accessed on 12 July 2021).
